# Greater Well-Being in More Physically Active Cancer Patients Who Are Enrolled in Supportive Care Services

**DOI:** 10.1177/1534735420921439

**Published:** 2020-05-26

**Authors:** Maíra Tristão Parra, Naghmeh Esmeaeli, Jordan Kohn, Brook L. Henry, Stephen Klagholz, Shamini Jain, Christopher Pruitt, Daniel Vicario, Wayne Jonas, Paul J. Mills

**Affiliations:** 1 University of California San Diego, La Jolla, CA, USA; 2 Samueli Integrative Health Programs, H&S Ventures, Alexandria, VA, USA; 3 Georgetown University, Washington, DC, USA

**Keywords:** physical activity, cancer care facilities, quality of life, biomarkers, cross-sectional

## Abstract

**Background:** Cancers are one of the leading causes of mortality worldwide. Cancer patients are increasingly seeking integrative care clinics to promote their health and well-being during and after treatment. **Aim:** To examine relationships between physical activity (PA) and quality of life (QoL) in a sample of cancer patients enrolling in integrative care in a supportive care clinic. Also, to explore circulating inflammatory biomarkers and heart rate variability (HRV) in relationship to PA and QoL. **Methods:** A cross-sectional design of adult patients who sought care in the InspireHealth clinic, Vancouver, British Columbia, Canada. Patients with complete PA data (n = 118) answered psychosocial questionnaires, provided blood samples, and received HRV recordings before enrollment. Patients were stratified into “less” versus “more” active groups according to PA guidelines (150 minutes of moderate or 75 minutes of vigorous PA or an equivalent combination). **Results:** Breast (33.1%) and prostate (10.2%) cancers were the most prevalent primary diagnoses. Patients engaging in more PA reported better physical (*U* = 1265.5, *P* = .013), functional (*U* = 1306.5, *P* = .024), and general QoL (*U* = 1341, *P* = .039), less fatigue (*U* = 1268, *P* = .014), fewer physical cancer-related symptoms (*U* = 2.338, *P* = .021), and less general distress (*U* = 2.061, *P* = .021). Between PA groups, type of primary cancer diagnosis differed (χ^2^ = 41.79, *P* = .014), while stages of cancer did not (χ^2^ = 3.95, *P* = .412). Fewer patients reported depressed mood within the more active group (χ^2^ = 6.131, *P* = .047). More active patients were also less likely to have ever used tobacco (χ^2^ = 7.41, *P* = .025) and used fewer nutritional supplements (χ^2^ = 39.74, *P* ≤ .001). An inflammatory biomarker index was negatively correlated with vigorous PA (*r*_s_ = −0.215, *P* = .022). Multivariable linear regression (*R*^2^ = 0.71) revealed that age (β = 0.22; *P* = .001), fatigue (β = −0.43; *P* ≤ .001), anxiety (β = −0.14; *P* = .048), and social support (β = 0.38; *P* = .001) were significant correlates of QoL.

## Background

Cancer is a generic term for a large group of diseases characterized by abnormal growth of cells, which can invade adjoining parts of the body and spread to other organs.^1^ Its incidence and mortality have been growing worldwide, and in 2018, new cases were estimated at 18.1 million, causing 9.6 million deaths.^2^ According to the World Health Organization (WHO), cancers rank among the top 20 disease-related causes of mortality worldwide, specifically tracheal, bronchial, and lung cancers (6th); liver cancer (16th); colorectal cancers (17th); and stomach cancers (18th).^3^ Among men, lung cancer is the most prevalent type and a leading cause of cancer deaths, whereas breast cancer is the most prevalent and lethal among women.^2^ In Canada, specifically, cancers (ie, malignant neoplasms) are currently the leading cause of death, surpassing mortality due to heart disease and causing more than 79 000 deaths in 2017.^4^

Biomedical advances have improved our understanding of the etiology of various cancers, as well as the development of effective treatments and prevention strategies. Yet, despite these efforts, cancer mortality continues to increase, highlighting the importance of prevention and risk factor mitigation. To that end, the WHO published the 2013 Global Action Plan for the prevention and control of noncommunicable diseases (NCDs), wherein a key objective was “to reduce modifiable risk factors for NCDs and underlying social determinants through the creation of health-promoting environments.”^5^ Strategies to prevent cancers and other NCDs include increasing physical activity (PA) levels, reducing alcohol and tobacco use, and promoting healthy diets.^5^ While prevention is essential, patients with a cancer diagnosis typically receive intensive and invasive treatments, including surgery, radiation therapy, chemotherapy, immunotherapy, and hormone therapy, which often have profound off-target effects on the body and brain that affect quality of life (QoL) through various mechanisms.^6^ Cancer and cancer treatments can cause physical discomfort, psychological distress, and a vast number of side effects, including pain, nausea and vomiting, fatigue, and sleep disturbances.^6^ Thus, strategies to decrease and alleviate such symptoms, and to promote and maintain general well-being and QoL for patients are critically needed.

Complementary and integrative medicine (CIM) treatments, including exercise, yoga/Tai Chi, and meditation, as an adjuvant to conventional oncology treatment may have beneficial impacts on psychological distress, anxiety, pain, fatigue, and sleep disturbances, leading to improved QoL in cancer patients.^7^ The mechanisms by which CIM interventions, particularly exercise, improve behavioral comorbidities in cancer patients are diverse, but likely involve modulatory effects on neuroimmune and neuroendocrine pathways associated with cancer and/or cancer treatment.^8,9^ Cancer-related fatigue (CRF), for instance, frequently co-occurs with elevated peripheral biomarkers of inflammation, including interleukin-6 (IL-6) and tumor necrosis factor-α (TNF-α), which can feedback into the central nervous system, thereby altering mood and cognition.^10^ Prior studies also indicate that low heart rate variability (HRV), a physiological marker of autonomic dysregulation and catecholamine (ie, neuroendocrine) signaling, is associated with both CRF and greater plasma levels of IL-6 in cancer survivors.^11,12^ Importantly, yoga^12^ and exercise interventions in cancer patients have been shown to decrease circulating TNF-α levels,^13^ and may improve the autonomic nervous system (ANS) regulation.^14^ Furthermore, persistent low-grade inflammation promotes deleterious cancer processes, including angiogenesis, metastasis, chemoresistance, and survival,^15,16^ which occur in part through nuclear factor-κβ (NF-κβ)-mediated gene regulation of processes associated with apoptosis suppression, angiogenesis, tumor migration, metastasis, and resistance to chemotherapy and radiation treatment.^17,18^ In addition, key inflammatory cytokines, such as (IL-1 and TNF-α, activate NF-κβ in cancer cells and may mediate cancer progression,^19,20^ and ANS imbalance is related to survival in a number of cancer populations.^21,22^

Initiating or continuing to engage in traditional, positive health behaviors, such as maintaining adequate levels of PA, may enhance QoL,^23^ reduce fatigue, and improve depressive symptoms that are commonly associated with cancer diagnosis.^24^ Moreover, a recent review highlighted the cellular and molecular mechanisms by which physical exercise can mitigate cancer progression through direct effects on tumor intrinsic factors, including growth rate, metastasis, tumor metabolism, and immunogenicity, as well as regulation of tumor growth through systemic processes, and improved cancer treatment efficacy.^25^

The Society of Integrative Oncology defines integrative oncology as “a patient-centered, evidence-informed field of cancer care that utilizes mind and body practices, natural products, and/or lifestyle modifications from different traditions alongside conventional cancer treatments. Integrative oncology aims to optimize health, QoL, and clinical outcomes across the cancer care continuum and to empower people to prevent cancer and become active participants before, during, and beyond cancer treatment.”^26^ The society recommends meditation, yoga, and relaxation with imagery to be routinely used for anxiety and mood disorders (evidence grade A), while stress management, yoga, massage, music therapy, energy conservation, and meditation are recommended for stress reduction, anxiety, depression, fatigue, and QoL (evidence grade B) of breast cancer patients.^27^ While the Society does not explicitly include PA in its guidelines, exercise programs are a common feature of integrative oncology practices globally.^28,29^

In the present study, we sought to describe the characteristics of cancer patients who seek CIM in combination with conventional oncology treatments. Furthermore, by comparing patients according to PA and characterizing relationships between PA, QoL, inflammatory biomarkers, and autonomic balance, this study provides insights into factors associated with QoL in cancer patients. We hypothesized that patients who were more physically active postdiagnosis would report better QoL, less fatigue, better psychological outcomes, and be more likely to engage in healthful behaviors. Secondarily, we hypothesized that more physically active patients would have lower levels of pro-inflammatory biomarkers and increased HRV, a measure of autonomic balance.

## Methods

The present study is a cross-sectional analysis of a cohort of cancer patients at the time of enrollment in a supportive care clinic.

### Setting

This study was conducted in a supportive care clinic called the InspireHealth Clinic, located in Vancouver, Canada. This is a physician-led, comprehensive cancer survivorship clinical care center providing integrative modalities, patient empowerment techniques, and lifestyle management approaches. The clinic staff is composed of family physicians and other professionals (including a registered holistic nutritionist, kinesiologist and exercise therapist, registered clinical counselor, integrated bodyworkers, and registered massage therapist).^30^ The InspireHealth foundations of health include sleep, rest and relaxation, exercise, healthy eating, avoidance of toxins, stress reduction, emotional connection, body-mind awareness, personal autonomy, joy and laughter, spiritual connection, hope, and will to live. Patients received their standard treatment at regional hospitals or branches of the British Columbia Cancer Agency.

### Participants

Participants were patients enrolling in InspireHealth Clinic services. Inclusion criteria were to be at least 18 years; diagnosed with any type and stage of cancer; to be enrolling for the first time in the clinic; to have a performance status level ≤2 assessed by the Eastern Cooperative Oncology Group Performance Status^31^ to ensure that patients could adequately participate, as this tool describes the patient’s level of physical functioning and the ability to care for themselves. Participants had to be able and willing to participate in the study; able to provide informed consent; able to read and write in English; able and willing to answer the questionnaires and to provide blood samples. We analyzed data of participants who had PA data available.

### Data Sources, Measurement, and Procedures

A study coordinator was responsible for patient recruitment and consenting, overseeing participants while completing the questionnaires, and managing participants’ files. When enrolling in the clinic, potential participants were told about the study, and if interested, the study coordinator contacted them by telephone or in person. They met with the study coordinator before their first physician visit, where they received comprehensive information about the study. All participants who met the eligibility criteria and agreed to participate signed the informed consent. Study numbers were assigned to participants for deidentification. The institutional review boards at Western Institutional Review Board (WIRB) approved this study with protocol number 20132240.

Participants completed all questionnaires during their clinic visit. The first part of the questionnaire captured self-reported sociodemographic characteristics, self-reported information about their cancer diagnosis, disease stage, treatment, family history of cancer, history of depression and anxiety, history of addiction, tobacco, and alcohol use. Participants also reported the use of medications and supplements (dose, purpose, and who prescribed it) and types of complementary and alternative medicine (CAM) therapies experienced. QoL was assessed with the Functional Assessment of Cancer Therapy–General (FACT-G) scale.^32^ This 29-item questionnaire is a validated and reliable tool that evaluates multiple dimensions of the QoL (physical, functional, emotional, and social well-being) during the previous week. Perceived level of social support was measured through the 19-item Medical Outcomes Study Social Support Survey (MOS-SSS),^33^ which quantifies overall support, as well as subscales assessing emotional/informational support, tangible support, positive interaction, and affection. We assessed anxiety and depression with the 14-item Hospital Anxiety and Depression Scale (HADS),^34^ which consists of 2 subscales ranging from 0 (no distress) to 21 (maximum distress). Based on suggested cutoffs, we classified participants as asymptomatic (≤7), borderline (8-10), or clinical cases (≥11). The FACT–Fatigue subscale was used to assess fatigue, which is a 13-item questionnaire previously validated in cancer patients.^35^ The items refer to the symptoms experienced in the past 7 days, and participants respond with 0 (not at all) to 4 (very much) how much they agree with the statements. Cancer-related symptoms were assessed with the Memorial Symptom Assessment Inventory–short form (MSAS-sf),^36^ which rates symptom distress associated with 26 physical symptoms, as well as the frequency of 4 psychological symptoms during the past week (eg, sadness, worry, irritability, nervousness). From the MSAS-sf assessment, subscales for physical and psychologic symptoms, a global distress index, and a total score were derived.

Patients reported their PA using a modified version of the Godin Leisure-Time Exercise Questionnaire (GLTEQ),^37^ which captured how many minutes per week they engaged in mild, moderate, and strenuous leisure PA. Patients reported their PA before and after the cancer diagnosis, and postdiagnosis PA was adopted to dichotomize the sample. Mild PA was described as activities requiring minimal effort, causing no perspiration (eg, easy walking, yoga, golf); moderate PA was defined as those not exhausting with light sweat (eg, fast walking, tennis, easy bicycling, easy swimming); and strenuous PA was described as those when the heart beats rapidly, causing sweat (eg, running, aerobic classes, vigorous swimming, or bicycling). The level of PA (more or less active), according to weekly recommendations of 150 minutes of moderate or 75 minutes of vigorous PA, or an equivalent combination,^38^ was our key independent variable. Because evidence suggests that higher intensities of PA (eg, moderate to vigorous) may have stronger effects on cancer outcomes than total PA volume per se, total weekly time spent engaging in PA at each intensity was also used as an outcome measure in our exploratory biomarkers analyses (see below).

To assess inflammatory biomarkers, we collected a venous blood sample at the regional LifeLabs clinic at the location closest to each participant’s residence. A trained and certified phlebotomist drew 10 mL of nonfasting blood. Whole blood was centrifuged for plasma and frozen at −80°F. Samples were shipped on dry ice to the University of California, San Diego (UCSD) Clinical Research Biomarker Laboratory, and assayed via Meso Scale Discovery (MSD) Systems (Rockville, MD). The enzyme-linked immunosorbent assay (ELISA)-based platform allows for simultaneous assays of multiple analytes. We determined circulating levels of C-reactive protein (CRP), IL-6, TNF-α, vascular endothelial growth factor (VEGF), and IL1ra (IL-1 receptor antagonist).^39,40^ Intra- and interassay coefficients were <7% (Table 1).

**Table 1.** Intra- and Inter-Assays CVs for Biomarkers.

	Intra-Assay CV (%)	Inter-Assay CV (%)
CRP	3.3	7.4
IL-6	7.6	>10
TNF-α	5.2	>10
VEGF	5.9	13.4
IL1ra	3.9	12.2

Abbreviations: CV, coefficient value; CRP, C-reactive protein; IL-6, interleukin 6; TNF-α, tumor necrosis factor-α; VEGF, vascular endothelial growth factor; IL1ra, interleukin-1 receptor antagonist.

After completing the questionnaires, participants were asked to sit alone either in the living room area of the clinic or in the research coordinator’s office for 10 minutes to record HRV, which is a noninvasive measure of beat-to-beat intervals of the heart rate (R-R intervals)—a dynamic measure of biological system function that provides information on ANS activity. ANS regulation is increasingly recognized as an indicator of cardiovascular health and is a predictor of heart-related morbidity and mortality.^41^ We used the Equivital EQ02 Life Monitor to assess HRV, which is a multiparameter ambulatory monitoring system equipped with a 2-lead electrocardiogram (ECG) system designed to record and transmit real-time mobile physiological data. The EQ02 system includes the Sensor Electronics Module, a small, lightweight sensor, and EQ02 sensor belts that can be worn for extended periods under clothing. Participants wore the monitors for a 10-minute testing session (divided into acclimation and rest phases of 5 minutes each), according to the methods of the Task Force of the European Society of Cardiology and the North American Society of Pacing and Electrophysiology.^41^ The HRV examined in this article is from both periods, acclimation and, rest time.

Heart rate variability data were analyzed using the VivoSense software platform (VivoSense, Inc, Newport Beach, CA). Digitized ECG data were evaluated to detect the R-wave peaks of the QRS complex. The power spectrum density of the HRV signal was assessed using the nonparametric Welch periodogram method with Fast Fourier Transform.^42^ We utilized a multistep process to identify and remove signal artifacts. First, the beat-to-beat ECG waveform was visually inspected, and missing, or unidentified R-peaks were manually relabeled. RR interval artifacts were subsequently removed with linear spline interpolation. Third, an automated VivoSense artifact marking algorithm was also applied to identify and remove ectopic beats and spurious heart rate (excluding heart rate above 220 or below 30 beats per minute) before HRV data output. Measures include the rMSSD (the square root of the mean squared differences of successive NN intervals), a time-domain measure of the short-term variation that estimate high-frequency variations in heart rate^41^; and the LF/HF ratio (low-frequency/high-frequency ratio), a frequency-domain measure, as well as heart rate and respiratory rate.

### Statistical Analysis

Distribution of variables was checked using the Shapiro-Wilk’s normality test. We calculated means and standard deviations for continuous variables, and absolute values and frequencies for categorical ones. Group comparisons (more active vs less active) were conducted using independent *t* tests or Mann-Whitney *U* and χ^2^ tests. We conducted a factorial analysis of biomarkers related to inflammation (CRP, IL-6, TNF-α, VEGF, IL1ra), to mitigate multicollinearity and reduce dimensionality, thereby creating an inflammatory index whereby higher values indicate greater circulating biomarker concentrations.^39^

As additional exploratory analyses, we investigated relationships between inflammation biomarkers^43^ and QoL^23^ with different PA intensities through Spearman’s correlations. Finally, we conducted a hierarchical multivariable regression analysis with QoL (total score) as the dependent variable. All tests were 2-tailed. The results were considered significant at the *P*< .05 level. We performed all analyses using SPSS (version 26.0) software package (IBM, Armonk, NY).

## Results

### Sociodemographic and Health-Related Characteristics

Patients (N = 162) were enrolled from December 2014 until April 2016 (Table 2). From these, 118 participants (69.5% female; aged 56.6 ± 11.3 years) with complete PA data composed our sample. Based on the American Cancer Society (ACS)’s PA guidelines, 53 patients (44.9%) met the criteria for sufficient PA, whereas 65 patients (55.1%) did not. Table 3 contains the clinical characteristics of the sample, stratified by activity level. Breast cancer was the most prevalent primary cancer diagnosis, affecting 33.1% of participants, followed by prostate cancer (10.2%), ovarian cancer, and lymphoma (9.3%). Regarding the stage of cancer, patients were roughly equally distributed across stages 1 to 4. Importantly, cancer stages did not differ between the less and more active groups (χ^2^ = 3.95, *P* = .41). The most common cancer treatments before enrolling in the InspireHealth clinic were classified as “other” (54.4%), followed by onetime surgery (21.1%), and >1 chemotherapy treatment (11.4%). Patients provided a qualitative description of which “other” therapies they had undergone, and no group differences in the prevalence of “other” therapeutic modalities were observed (χ^2^ = 12.18, *P* = .431). Within the “other” therapies, patients identified: (1) planned, near-term cancer treatment (less vs more active: 28.6% vs 22.2%); received (2) steroid treatment (2.9% vs none); (3) combination of surgery and radiotherapy (14.3% vs 11.1%); (4) surgery and hormone therapy (2.9% vs none); (5) surgery and chemotherapy (20% vs 7.4%); (6) surgery and cystoscopy (none vs 3.7%); (7) combination of surgery, radiotherapy, and chemotherapy (11.4% vs 14.8%); (8) combination of surgery, radiotherapy, and hormone therapy (none vs 3.7%); (9) chemotherapy and radiotherapy (2.9% vs 7.4%); (10) chemotherapy and a transplant (5.7% vs none); (11) chemotherapy and Gerson therapy (none vs 3.7%); (12) radiation and hormone therapy (none vs 3.7%); and (13) no therapy or information available (11.4% vs 22.2%; data not shown in tables). Participants in the more active group reported a significantly lower incidence of history of depression than the less active group (χ^2^ = 10.64, *P* = .031). Many participants (31.4%) reported having a history of greater anxiety than the “average person,” but the prevalence did not differ between groups (χ^2^ = 2.02, *P* = .731).

**Table 2.** Self-Reported Sociodemographic Characteristics According to the Amount of Physical Activity (Reported as Means ± SDs or Absolute and Relative [%] Values).

	Total	n	Less Active	n	More Active	n	Test Statistics^a^	*P*	Effect Sizes
Age (years)	56.6 ± 11.3	118	55.5 ± 11.0	65	58.0 ± 11.6	53	1475	.180	−0.12^b^
Sex		118		65		53	3.770	.052	−0.17^c^
Male	36 (30.5)		15 (23.1)		21 (39.6)				
Female	82 (69.5)		50 (76.9)		32 (60.4)				
Ethnicity		118		65		53	6.55	.364	0.20^d^
Asian/Pacific Islander	18 (15.3)		11 (16.9)		7 (13.2)				
African/Black North American	1 (0.80)		1 (1.5)		-				
Caucasian	87 (73.7)		44 (50.6)		43 (81.1)				
East Indian	2 (1.7)		1 (1.5)		1 (1.9)				
Hispanic/Latino	2 (1.7)		2 (3.1)		—				
Native American/Native Alaskan	1 (0.8)		1 (1.5)		—				
Other	7 (5.9)		2 (3.8)		2 (3.8)				
Education level		118		65		53	9.53	.482	0.26^d^
Some high school	1 (0.8)		1 (1.5)		—				
High school graduate	5 (4.2)		2 (3.1)		3 (5.7)				
Some college	23 (19.5)		13 (20)		10 (18.9)				
College diploma	11 (9.3)		4 (6.2)		7 (13.2)				
2-year college graduate	9 (7.6)		3 (4.6)		6 (11.3)				
4-year college graduate	19 (16.1)		11 (16.9)		8 (15.1)				
Attended university	4 (3.4)		3 (4.6)		1 (1.9)				
University degree	11 (9.3)		8 (12.3)		3 (5.7)				
Some graduate school	1 (0.8)		1 (1.5)		—				
Postgraduate degree	33 (28)		18 (27.7)		15 (28.3)				
Partner status		117		64		53	6.46	.167	0.23^d^
Single, never married	20 (17.1)		10 (15.6)		10 (18.9)				
Separated/divorced	21 (17.9)		10 (15.6)		11 (20.8)				
Widowed	2 (1.7)		2 (3.1)		—				
Living with a partner	12 (10.3)		10 (15.6)		2 (3.8)				
Married living with a spouse	62 (53)		32 (50)		30 (56.6)				
Children		116		64		52	1.24	.265	−0.10^c^
No	47 (40.5)		23 (35.9)		24 (46.2)				
Yes	69 (59.5)		41 (64.1)		28 (53.8)				

a Test statistics are reported as *U* for continuous variables and as χ^2^ for categorical variables.

b Effect size reported as *r*.

c Effect sizes reported as Phi.

d Effect sizes reported as Cramér’s *V*.

**Table 3.** Self-Reported Health-Related Characteristics of the Total Sample and by the Amount of Physical Activity (Reported as Absolute and Relative [%] Values).

	Total	n	Less Active	n	More Active	n	χ^2^	*P*	Cramér’s *V*
Primary cancer diagnosis		118		65		53	41.79	.014	0.54
Bladder	1 (0.8)		—		1 (1.9)				
Brain	4 (3.4)		2 (3.1)		2 (3.8)				
Breast	39 (33.1)		25 (38.5)		14 (26.4)				
Cervical	1 (0.8)		1 (1.5)		—				
Colon	8 (6.8)		6 (9.2)		2 (3.8)				
Colorectal	2 (1.7)		1 (1.5)		1 (1.9)				
Endometrial	4 (3.4)		1 (1.5)		3 (5.7)				
Epithelioid	1 (0.8)		—		1 (1.9)				
Esophageal	2 (1.7)		1 (1.5)		1 (1.9)				
Gastrointestinal	1 (0.8)		1 (1.5)		—				
Laryngeal	1 (0.8)		—		1 (1.9)				
Leiomyosarcoma	1 (0.8)		1 (1.5)		—				
Leukemia	2 (1.7)		2 (3.1)		—				
Lung	3 (2.5)		2 (3.1)		1 (1.9)				
Lymphoma	11 (9.3)		4 (6.2)		7 (13.2)				
Melanoma	1 (0.8)		1 (1.5)		—				
Multiple myelomas	1 (0.8)		1 (1.5)		—				
Ovarian	11 (9.3)		9 (13.8)		2 (3.8)				
Pheochromocytoma	1 (0.8)		1 (1.5)		—				
Prostate	12 (10.2)		1 (1.5)		11 (20.8)				
Rectal	2 (1.7)		2 (3.1)		—				
Skin	1 (0.8)		—		1 (1.9)				
Thyroid	2 (1.7)		1 (1.5)		1 (1.9)				
Tongue	1 (0.8)		—		1 (1.9)				
Uterine	5 (4.2)		2 (3.1)		3 (5.7)				
Cancer stage		93		55		38	3.95	.412	0.20
Stage 1	18 (19.4)		10 (18.2)		8 (21.1)				
Stage 2	20 (21.5)		11 (20)		9 (23.7)				
Stage 3	23 (24.7)		17 (30.9)		6 (15.8)				
Stage 4	24 (25.8)		14 (25.5)		10 (26.3)				
Unknown	8 (8.6)		3 (5.5)		5 (13.2)				
Cancer treatment before admission		114		64		50	3.21	.817	0.16
Chemotherapy (1×)	5 (4.4)		2 (3.1)		3 (6)				
Chemotherapy (>1)	13 (11.4)		9 (14.1)		4 (8)				
Surgery (1×)	24 (21.1)		12 (18.8)		12 (24)				
Surgery (>1)	3 (2.6)		2 (3.1)		1 (2)				
Radiation (1×)	3 (2.6)		1 (1.6)		2 (4)				
Radiation (>1)	4 (3.5)		3 (4.7)		1 (2)				
Other	62 (54.4)		35 (54.7)		27 (54)				
Family cancer history		117		65		52	4.64	.200	0.19
No history	21 (17.9)		9 (13.8)		12 (23.1)				
Close family member	19 (16.2)		11 (16.9)		8 (15.4)				
Parent with cancer	23 (19.7)		10 (15.4)		13 (25)				
Multiple family members	54 (46.2)		35 (53.8)		19 (36.5)				
Self-reported history of depression		118		65		53	10.64	.031	0.28
Much less than the average person	35 (29.7)		20 (30.8)		15 (42.9)				
Less than average	21 (17.8)		7 (10.8)		14 (26.4)				
Like average person	32 (27.1)		16 (24.6)		16 (30.2)				
More than average	27 (22.9)		19 (29.2)		8 (29.6)				
Much more than the average person	3 (2.5)		3 (4.6)		—				
Self-reported history of anxiety		118		65		53	2.02	.731	0.12
Much less than the average person	29 (24.6)		15 (23.1)		14 (26.4)				
Less than average	16 (13.6)		10 (15.4)		6 (11.3)				
Like average person	31 (26.3)		16 (24.6)		15 (28.3)				
More than average	37 (31.4)		20 (30.8)		17 (32.1)				
Much more than the average person	5 (4.2)		4 (6.2)		1 (1.9)				

Health-related behaviors of the participants are shown in Table 4. Most patients (89.7%) did not have a history of addiction and never smoked (94.9%), though all current and former smokers were contained within the less active group (χ^2^ = 7.41, *P* = .025). Occasional alcohol consumption (once a week or less) was a prevalent behavior (49.2%) among all patients. Self-reported engagement in leisure PA was, on average, 283 min/week, and most time was spent engaging in activities of mild (135 min/week) to moderate (114 min/week) intensity. In general, participants had an extensive regimen of medications. Analgesic and chemotherapy drugs were the most prevalent (15.6% and 14.5%, respectively), and physicians prescribed 56.7% of the medications. Most participants (77.4%) reported the use of some supplements. Less active patients used significantly more supplements than the more active patients (66.9% vs 33.1%, χ^2^ = 39.74, *P* < .001), and vitamin supplements were prevalent (37.2%). Naturopathic physicians prescribed more supplements (12.2%) than other physicians (8.1%). Participants had a previous engagement with complementary or alternative therapies, mostly massage (75.2%) and yoga (66.1%) and acupuncture (59.6%).

**Table 4.** Self-Reported Health-Related Behaviors of the Total Sample and Divided by the Amount of Physical Activity (Reported as Means ± SDs or Absolute and Relative (%) Values).

	Total	n	Less Active	n	More Active	n	Test Statistics^a^	*P*	Effect Sizes
History of addiction		117		65		52	2.67	.102	0.15^b^
No	105 (89.7)		61 (93.8)		44 (84.6)				
Yes	12 (10.3)		4 (6.2)		8 (15.4)				
Tobacco use		118		65		53	7.41	.025	0.20^c^
Never	112 (94.9)		59 (90.8)		53 (100)				
Occasionally (1×/week or less)	1 (0.8)		1 (1.5)		—				
Every day or almost every day	5 (4.2)		5 (7.7)		—				
Alcohol use		117		64		53	5.14	.076	0.21^c^
Never	42 (35.6)		25 (39.1)		17 (32.1)				
Occasionally (1×/week or less)	58 (49.2)		34 (53.1)		24 (45.3)				
Every day or almost every day	17 (14.4)		5 (7.8)		12 (22.6)				
Physical activity before diagnosis (min/week) (GLTEQ)		118		65		53			
Vigorous	110.97 ± 154.17		94.65 ± 18.40		130.99 ± 21.99		1379	.055	−0.17^d^
Moderate	167.54 ± 189.17		171.94 ± 23.77		162.15 ± 25.79		1688	.854	−0.01^d^
Mild	161.80 ± 216.70		166.15 ± 31.92		156.46 ± 21.31		1513	.255	−0.10^d^
Total	440.31 ± 400.25		432.74 ± 58.30		449.60 ± 40.81		1386	.069	−0.16^d^
Physical activity after diagnosis (min/week) (GLTEQ)		118		65		53			
Vigorous	33.67 ± 70.25		3.92 ± 17.24		70.14 ± 90.99		929	<.001	−0.50^d^
Moderate	114.08 ± 130.60		29.36 ± 47.01		217.97 ± 125.20		191	<.001	−0.78^d^
Mild	135.97 ± 160.69		131.19 ± 177.49		141.84 ± 138.79		1504	.234	−0.10^d^
Total	283.71 ± 232.19		164.47 ± 174.34		429.95 ± 210.54		381	<.001	−0.66^d^
Reported use of medications		118		65		53	42.91	.076	0.34^c^
Acid reducer	17 (5.9)		10 (5.5)		7 (6.5)				
Antidiarrheal	2 (0.7)		1 (0.6)		1 (0.9)				
Antiemetic	14 (4.8)		9 (5.0)		5 (4.6)				
Antihistamine	6 (2.1)		4 (2.2)		2 (1.9)				
Antihypertensive	23 (8.0)		14 (7.7)		9 (8.3)				
Antimigraine	1 (0.3)		1 (0.6)		—				
Antiviral	4 (1.4)		2 (1.1)		2 (1.9)				
Benign prostatic hypertensive	3 (1.0)		—		3 (2.8)				
Bile agent acid	1 (0.3)		1 (0.6)		—				
Bronchodilator	5 (1.7)		4 (2.2)		1 (0.9)				
Chemotherapy	42 (14.5)		28 (15.5)		14 (13.0)				
Anal fissure treatment	1 (0.3)		—		1 (0.9)				
Cholesterol reducer	8 (2.8)		6 (3.3)		2 (1.9)				
Hematopoietic agent	1 (0.3)		1 (0.6)		—				
Hormone therapy	25 (8.7)		14 (7.7)		11 (10.2)				
Laxative	2 (0.7)		2 (1.1)		—				
Mucolytic agent	1 (0.3)		—		1 (0.9)				
Muscarinic antagonist	2 (0.7)		1 (0.6)		1 (0.9)				
Muscle relaxant	2 (0.7)		1 (0.6)		1 (0.9)				
Sleeping aid/calming	23 (8.0)		16 (8.8)		7 (6.5)				
Steroid	17 (5.9)		10 (5.5)		7 (6.5)				
Stimulant	2 (0.7)		1 (0.6)		1 (0.9)				
Analgesic	45 (15.6)		26 (14.4)		19 (17.6)				
Triptan	2 (0.7)		2 (1.1)		—				
Ulcerative colitis treatment	1 (0.3)		—		1 (0.9)				
Xanthine oxidase inhibitor	1 (0.3)		—		1 (0.9)				
Antidiabetic	4 (1.4)		4 (2.2)		—				
Antipsychotic	2 (0.7)		2 (1.1)		—				
Antibiotics	4 (1.4)		—		4 (3.7)				
Anticoagulant	1 (0.3)		—		1 (0.9)				
Anticonvulsant	3 (1.0)		2 (1.1)		1 (0.9)				
Antidepressant	24 (8.3)		19 (10.5)		5 (4.6)				
Total	289 (100)		181 (62.6)		108 (37.4)				
Who prescribed the drugs		118		65		53	14.44	.001	0.22^c^
Physician	164 (56.7)		88 (48.6)		76 (70.4)				
Self	4 (1.4)		2 (1.1)		2 (1.9)				
Missing information	121 (41.9)		91 (50.3)		30 (24.8)				
Reported use of supplements		118		65		53	39.74	<.001	0.29^c^
Amino acids	9 (2.3)		5 (1.9)		4 (3.1)				
Fatty acids	23 (5.9)		14 (5.3)		9 (6.9)				
Homeopathy	1 (0.3)		—		1 (0.8)				
Melatonin	13 (3.3)		10 (3.8)		3 (2.3)				
Other	12 (3.1)		11 (4.2)		1 (0.8)				
Plant extract	55 (14)		42 (76.4)		13 (23.6)				
Probiotics	10 (2.5)		7 (2.7)		3 (2.3)				
Vitamins	146 (37.2)		95 (36.1)		51 (39.2)				
Animal source	5 (1.3)		—		5 (3.8)				
Antioxidants	8 (2.0)		5 (1.9)		3 (2.3)				
Ayurvedic supplement	15 (3.8)		10 (3.8)		5 (3.8)				
Bone health	5 (1.3)		1 (0.4)		4 (3.1)				
Cannabis	3 (0.8)		3 (1.1)		—				
Chinese medicine	13 (3.3)		13 (4.9)		—				
Elements	61 (15.5)		38 (14.4)		23 (17.7)				
Enzymes	14 (3.6)		9 (3.4)		5 (35.7)				
Total	393 (100)		263 (66.9)		130 (33.1)				
Who prescribed the supplements		118		65		53	78.44	<.001	0.44^c^
Physician	32 (8.1)		14 (5.3)		18 (13.8)				
Self	29 (7.4)		8 (3.0)		21 (16.2)				
Naturopath	48 (12.2)		16 (6.1)		32 (24.6)				
Chiropractor	5 (1.3)		5 (1.9)		—				
Nutritionist	2 (0.5)		—		2 (1.5)				
Pharmacist	3 (0.8)		2 (0.8)		1 (0.8)				
Missing information	274 (69.7)		218 (55.5)		56 (43.1)				
Types of CAM therapies									
Acupuncture	65 (59.6)	109	38 (63.3)	60	27 (55.1)	49	0.759	.384	−0.08^b^
Muscle relaxation exercises	36 (33)	109	19 (31.7)	60	17 (34.7)	49	0.112	.738	0.03^b^
Yoga	72 (66.1)	109	39 (65)	60	33 (67.3)	49	0.066	.797	0.02^b^
Visualization exercises	43 (39.4)	109	22 (36.7)	60	21 (42.9)	49	0.433	.511	0.06^b^
Stress reduction exercises	30 (27.5)	109	16 (26.7)	60	14 (28.6)	49	0.049	.825	0.02^b^
Massage	82 (75.2)	109	48 (80)	60	34 (69.4)	49	1.630	.202	0.12^b^
Hypnosis	22 (20.2)	109	13 (21.7)	60	9 (18.4)	49	0.182	.669	0.04^b^
Naturopathic medicine	45 (41.3)	109	24 (40)	60	21 (42.9)	49	0.091	.763	0.02^b^
Dietary vitamin therapies	42 (38.5)	109	24 (40)	60	18 (36.7)	49	0.121	.727	0.03^b^
Other	31 (28.4)	103	22 (36.7)	56	9 (18.4)	47	4.438	.035	−0.20^b^

Abbreviations: GLTEQ, Godin Leisure-Time Exercise Questionnaire; CAM, complementary and alternative medicine.

a Test statistics are reported as *U* for continuous variables and as χ^2^ for categorical variables.

b Effect sizes reported as Phi.

c Effect sizes reported as Cramér’s *V*.

d Effect size reported as *r*.

### Physical Activity and Psychosocial Characteristics

Patients who were more physically active reported better QoL (*U* = 1341, *P* = .039), and greater physical (*U* = 1265.5, *P* = .013) and functional (*U =* 1306.5, *P* = .024) well-being. The prevalence of depressive symptoms was significantly lower among more active patients (χ^2^ = 6.13, *P* = .047). More active participants also reported less fatigue (*U* = 1268, *P* = .014), fewer physical symptoms related to cancer (*U* = 2.338, *P* = .021), and less generalized distress (*U* = 2.339, *P* = .021) compared with less active participants. Social, familial, or emotional well-being subscales did not differ between PA groups, nor did perceived social support or anxiety prevalence (Table 5).

**Table 5.** Self-Reported Psychosocial and Physical Characteristics of the Total Sample and by the Amount of Physical Activity (Reported as Means ± SDs or Absolute and Relative [%] Values).

	Total (n = 118)	Less Active (n = 65)	More Active (n = 53)	Test Statistics^a^	*P*	Effect Sizes
Quality of life (FACT-G)						
Physical well-being	21.56 ± 5.55	20.62 ± 5.65	22.72 ± 5.25	1265.5	.013	−0.22^b^
Social and family well-being	20.31 ± 5.62	20.17 ± 6.22	20.49 ± 4.83	1615	.559	−0.05^b^
Emotional well-being	16.08 ± 4.90	15.48 ± 4.58	16.81 ± 5.20	1410.5	.091	−0.15^b^
Functional well-being	18.25 ± 5.66	17.17 ± 5.72	19.58 ± 5.33	1306.5	.024	−0.20^b^
Total score	76.20 ± 15.47	73.43 ± 16.43	79.60 ± 13.59	1341	.039	−0.19^b^
Social support (MOS-SSS)						
Emotional/informational support	72.64 ± 24.30	69.47 ± 25.70	76.53 ± 22.05	1444	.130	−0.13^b^
Tangible support	72.56 ± 31.97	70.86 ± 34.23	74.64 ± 29.14	1674	.788	−0.02^b^
Affectionate support	78.96 ± 29.72	77.56 ± 31.79	80.66 ± 27.13	1650	.671	−0.03^b^
Positive social interaction support	77.40 ± 25.95	75.00 ± 27.40	80.38 ± 23.96	1560	.357	−0.08^b^
Overall support	74.47 ± 23.79	71.98 ± 25.54	77.50 ± 21.27	1504	.237	−0.10^b^
Anxiety (HADS)				0.175	.916	0.03^c^
Asymptomatic	72 (61)	39 (60)	33 (62.3)			
Borderline	22 (18.6)	13 (20)	9 (17)			
Clinical case	24 (20.3)	13 (20)	11 (20.8)			
Depression (HADS)				6.131	.047	0.22^c^
Asymptomatic	101 (85.6)	51 (78.5)	50 (94.3)			
Borderline	8 (6.8)	7 (10.8)	1 (1.9)			
Clinical case	9 (7.6)	7 (10.8)	2 (3.8)			
Fatigue (FACT)	17.49 ± 11.38	20.06 ± 12.10	14.33 ± 9.63	1268	.014	−0.22^b^
Symptoms associated with cancer (MSAS-sf)						
Physical symptoms	1.32 ± 0.43	1.40 ± 0.44	1.22 ± 0.39	2.338	.021	−0.21^b^
Psychological symptoms	1.47 ± 0.59	1.52 ± 0.61	1.40 ± 0.56	1.049	.296	−0.22^b^
Global distress index	1.40 ± 0.45	1.49 ± 0.47	1.30 ± 0.41	2.339	.021	−0.09^b^
Total score	1.32 ± 0.37	1.39 ± 0.39	1.25 ± 0.32	2.061	.042	−0.16^b^

Abbreviatons: FACT-G, Functional Assessment of Cancer Therapy–General scale; MOS-SSS, Medical Outcomes Study Social Support Survey; HADS, Hospital Anxiety and Depression Scale; FACT, Functional Assessment of Cancer Therapy–Fatigue subscale; MSAS-sf, Memorial Symptom Assessment Inventory–Short-form.

a Test statistics are reported as *U* for continuous variables and as χ^2^ for categorical variables.

b Effect size reported as *r*.

c Effect sizes reported as Cramér’s *V*.

In addition to classifying patients into less and more active groups based on ACS guidelines, we also assessed the amount of PA for all participants within each intensity level (eg, weekly minutes of mild, moderate, or vigorous activity) as a continuous measure. When exploring the relationship between QoL and PA, we found positive correlations for total time (*r*_s_ = 0.227, *P* = .014) and moderate-intensity PA (*r*_s_ = 0.291, *P* = .001) (Table 6). A hierarchical multivariable linear regression model was implemented to investigate the correlates of total QoL score (Table 7). The overall model explained 71.1% of the variability in our dependent variable, with age (β = 0.22; *P* = .001), fatigue (β = −0.43; *P* < .001), anxiety (β = −0.14; *P* = .048) and social support (β = 0.38; *P* = .001) as significant correlates of total QoL.

### Biomarkers

Heart rate was significantly lower in more physically active patients during the acclimation phase (*t* = 2.085, *P* = .039) and the resting period (*t* = 2.120, *P* = .036) of the HRV assessment. Across all other cardiopulmonary measures of health, differences between groups were not statistically significant.

After examining PA levels (more versus less active, according to ACS guidelines), we decided to explore relationships of PA intensities and inflammation, as prior studies have suggested that high intensities of exercise might have a differential impact on inflammation.^44-46^ Biomarkers related to inflammation and autonomic balance are reported in Tables 6 and 8. All 5 inflammatory biomarkers were entered into a factor analysis^39^ to reduce the number of analyses, which resulted in a single stable component (Eigenvalue = 2.075) that explained 41.9% of the variance and was used in subsequent analyses. The individual factor component scores were then used as a unitary inflammatory index for each patient. Vigorous PA was inversely associated with the inflammatory index (*r*_s_ = −0.215, *P* = .022). Heart rate was significantly lower in more physically active patients during the acclimation phase (*t* = 2.085, *P* = .039) and the resting period (*t* = 2.120, *P* = .036) of the HRV assessment. Across all other measures of health, differences between groups were not statistically significant.

**Table 6.** Associations of the Inflammatory Biomarker Index and Quality of Life With Different Physical Activity Intensities (Light, Moderate, Vigorous, and Total Time of Physical Activity).

	Total Time		Light		Moderate		Vigorous	
	*r*_s_	*P*e	*r*_s_	*P*	*r*_s_	*P*	*r*_s_	*P*
Inflammatory index	−0.150	.115	−0.029	.763	−0.171	.071	−0.215	.022
QoL (FACT-G)	0.227	.014	0.028	.761	0.291	.001	0.092	.323

Abbreviations: QoL, quality of life; FACT-G, Functional Assessment of Cancer Therapy–General scale.

**Table 7.** Hierarchical Multivariable Linear Regression Model Predicting the Quality of Life in Cancer Patients (n = 86).

	*R* ^2^	Adjusted *R*^2^	Δ*R*^2^	Unstandardized β (95% CI)	SE B	Standard Coefficients β	VIF	*P*
Step 1	0.131	0.110						
Constant				50.98 (30.46 to 71.50)	10.31			<.001
Age				0.48 (0.20 to 0.75)	0.14	0.36	1.005	.001
Sex				−1.35 (−8.52 to 5.82)	3.61	−0.04	1.005	.709
Step 2		0.496	0.388					
Constant				72.58 (56.03 to 89.12)	8.32			<.001
Age				0.30 (0.09 to 0.51)	0.10	0.22	1.055	.006
Sex				1.52 (−4.02 to 7.06)	2.78	0.04	1.057	.586
Cancer stage				0.002 (0.000 to 0.004)	0.001	0.13	1.076	.111
Fatigue level				−0.90 (−1.12 to −0.68)	0.11	−0.65	1.102	.000
Step 3		0.714	0.218					
Constant				64.26 (47.90 to 80.63)	8.22			<.001
Age				0.30 (0.13 to 0.47)	0.08	0.22	1.215	.001
Sex				−0.42 (−4.65 to 3.81)	2.12	−0.01	1.090	.843
Cancer stage				0.001 (0.000 to 0.003)	0.001	0.09	1.163	.130
Fatigue level				−0.59 (−0.78 to −0.40)	0.09	−0.43	1.417	<.001
Depression				−5.77 (−11.71 to 0.16)	2.98	−0.13	1.490	.056
Anxiety				−2.92 (−5.83 to −0.02)	1.45	−0.14	1.599	.049
Social support				0.23 (0.16 to 0.32)	0.04	0.38	1.204	<.001
Step 4		0.711	<0.001					
Constant				63.85 (47.11 to 80.58)	8.40			<.001
Age				0.30 (0.13 to 0.47)	0.08	0.22	1.216	.001
Sex				−0.31 (−4.65 to 4.03)	2.18	−0.009	1.130	.888
Cancer stage				0.001 (0.000 to 0.003)	0.001	0.09	1.180	.127
Fatigue level				−0.59 (−0.78 to −0.40)	0.09	−0.43	1.444	<.001
Depression				−5.69 (−11.70 to 0.32)	3.01	−0.13	1.508	.063
Anxiety				−2.96 (−5.90 to −0.02)	1.47	−0.14	1.615	.048
Social support				0.23 (0.15 to 0.31)	0.04	0.38	1.210	<.001
Physical activity				0.54 (−3.37 to 4.45)	1.96	0.01	1.110	.783

Abbreviations: CI, confidence interval; SE, standard error; VIF, variance inflation factor.

**Table 8.** Health-Related Biomarkers of the Total Sample and by the Amount of Physical Activity (Reported as Means ± SDs).

	Total	n	Less Active	n	More Active	n	Test Statistics	*P*	*r*
CRP (mg/L)	3.39 ± 4.23	112	3.77 ± 4.51	62	2.93 ± 3.83	50	1384	.163	−0.13
IL-6 (pg/mL)	1.33 ± 1.57	112	1.44 ± 1.54	62	1.20 ± 1.62	50	1260	.090	−0.16
TNF-α (pg/mL)	2.07 ± 1.51	112	2.18 ± 1.90	62	1.93 ± 0.81	50	1617	.933	−0.00
VEGF (pg/mL)	68.05 ± 70.43	112	68.71 ± 61.31	62	67.23 ± 81.08	50	1551	.648	−0.04
IL1ra (pg/mL)	420.76 ± 290.07	112	458.65 ± 300.84	62	373.29 ± 271.41	50	1310	.070	−0.17
Inflammatory index^a^	NA	112	.10737 ± .9341	62	−.13315 ± 10.706	50	1.269^b^	.207	0.12
HRV acclimation (5 minutes)		112		63		49			
Heart rate (bpm)	73.37 ± 9.38		74.98 ± 8.29		71.31 ± 10.34		2.085^b^	.039	0.19
Respiratory rate (bpm)	15.43 ± 4.69		15.86 ± 4.55		14.90 ± 4.87		1324	.198	−0.12
rMSSD (ms)	21.98 ± 17.25		22.95 ± 19.59		20.72 ± 13.75		1497	.785	−0.02
LFHF ratio	4.25 ± 5.39		3.51 ± 3.49		5.21 ± 7.06		1424	.483	−0.06
HRV rest (5 minutes)									
Heart rate (bpm)	73.49 ± 9.20		75.09 ± 8.32		71.43 ± 9.94		2.120^b^	.036	0.19
Respiratory rate (bpm)	15.56 ± 4.87		15.99 ± 4.68		14.99 ± 5.10		1317	.184	−0.12
rMSSD (ms)	21.98 ± 18.37		22.40 ± 19.97		21.45 ± 16.29		1515	.867	−0.01
LFHF ratio	5.10 ± 6.97		3.75 ± 3.26		6.84 ± 9.66		1442	.552	−0.05

Abbreviations: CRP, C-reactive protein; IL-6, interleukin 6; TNF-α, tumor necrosis factor-α; VEGF, vascular endothelial growth factor; IL1ra, interleukin-1 receptor antagonist; HRV, heart rate variability; bpm, beats per minute; rMSSD, square root of the mean squared differences of successful N-N intervals; ms, milliseconds; LFHF, low frequency/high frequency.

a The inflammatory index had an Eigenvalue = 2.075. Eigenvalues ≥1 represent that the factor explains more variance that an inflammatory marker alone.

b *t* statistic. All other test statistics are reported as (*U*).

## Discussion

The patients in this study were similar to other studies describing characteristics of cancer patients seeking CIM: predominantly middle-aged, white, and a large proportion affected by breast cancer.^47-51^ We identified different stages of cancer in our sample, demonstrating that regardless of disease stage, integrative care seems attractive to cancer patients. In Germany, a survey found that 75% of patients (229 respondents) with advanced breast cancer (including patients with adjuvant cancer, local recurrence, and primary metastasis) were interested in complementary practices.^52^ A more extensive German study, including 3411 patients from 339 centers, found that 46.4% of breast cancer patients (tumor stages varied from 1 to 4) were interested in integrative medicine.^47^ In California, 95% of 166 advanced gastric nonsurgical patients were involved in at least one type of integrative care approach while receiving care in a hospital.^48^ Moreover, a survey of hospitalized Canadian cancer patients found that 86% reported to have received complementary care in the past month, and 91% in the past year; their reasons for its use included a desire to “feel good,” to relieve symptoms and side effects, and mostly to improve their overall QoL.^51^ Although rates of CIM utilization vary across settings and populations, a large proportion of cancer patients seek complementary therapies and express interest in integrative care for their condition.

In assessing the health-related behaviors of our study population, smoking was rare (5%), and among the more physically active patients, none were smokers. Our smoking rates were much lower than a Canadian case-control study that investigated PA in cancer patients, with a 14% rate of current smokers and 29.1% nonsmokers,^53^ potentially indicating that CIM-seeking patients engage in fewer harmful health behaviors than cancer patients as a whole. In support of that hypothesis, we also found that alcohol consumption was particularly infrequent in our sample. Regarding PA, in general, participants engaged primarily in moderate- to mild-intensity activity after their cancer diagnosis. For a reference, both the ACS guidelines for cancer prevention^38^ and the Canadian guidelines for exercise during and post–cancer treatment,^54^ recommend at least 150 minutes of moderate PA per week for cancer prevention and improvement of QoL for those fighting cancer. Cancer patients experience barriers to exercise resulting from treatment-related side effects, lack of time, and fatigue.^55^ Despite these limitations, cancer patients should be encouraged to exercise in moderate to vigorous intensities when possible to benefit from the effectiveness of regular exercise to increase the QoL,^23,56^ muscular and aerobic fitness,^54^ to reduce fatigue, depression, anxiety, and sleep disturbances.^56^

Our sample reported taking a variety of medications, with chemotherapies and analgesics being the most prevalent. Also, 77.4% of the participants reported using some supplement, with vitamins, including single-type or multicomplex of vitamins, being most frequently used. These results are in concordance with other studies that also identified high rates of use of vitamins and supplements in cancer patients, varying from 67% to 91% of cancer patients.^48,49,51^ Interestingly, we observed that patients who were more physically active used half of the supplements used by their less-active peers. The more active patients tended to get their prescriptions from naturopathic physicians, which might suggest they more frequently seek treatment from naturopathic physicians. We hypothesize that greater QoL among more active patients could be related to these patients seeking less additional supplements for symptom management or improvement in well-being, and we suggest this hypothesis be tested in a future, longitudinal study.

In addition to supplements use, participants reported their previous use of CAM therapies. Massage, yoga, and acupuncture were most frequently utilized (75.2%, 66.1%, and 59.6%, respectively). We did not observe differences in the use of CAM therapies between groups. Compared with other studies, our sample was highly engaged in CAM therapies, which was expected, considering that our sample was selected by convenience from a pool of patients who were interested in enrolling in integrative therapies. By comparison, Liu and collaborators saw 39% use of manual therapies, including massage and acupressure,^48^ Frenkel and collaborators observed 10% use of massage therapy use and 16% of cancer patients engaged in Asian movement therapies, including yoga, tai chi, or Qi Gong, before enrolling in an integrative oncology clinic.^49^ A limitation of our analysis is that we queried the use of CAM therapies individually, and thus were unable to detect if patients were using multiple CAMs simultaneously.

We found that more physically active patients reported a better QoL than their less-active peers, specifically their physical and functional well-being. Others have reported similar associations in a case-control design,^53^ and the positive, causal effects of exercise interventions (eg, 6 months on an exercise program) on QoL in cancer patients has been confirmed by more robust study designs.^23,56^ More physically active patients also reported lower fatigue levels and fewer cancer-related physical symptoms, which aligns with prior reports of decreased fatigue among patients who exercised during their cancer treatment.^56^ However, another systematic review evaluated the effectiveness of exercise for cancer patient QoL and suggested that exercise does not perform better than standard care, though it does appear to reduce fatigue.^57^

There were fewer borderline and clinical cases (according to the suggested cut points) of depression in the more physically active group. We also observed fewer cases of anxiety in the more physically active group, although the prevalence differences for anxiety did not reach statistical significance between groups. While a handful of trials report positive associations and effects of PA on mental health in cancer patients,^58,59^ and that insufficient PA is associated with depression and anxiety,^60^ more robust evidence from systematic reviews of randomized controlled trials reported mild or insignificant changes for depression and anxiety.^56,61,62^

We found that perceived social support was superior among more active patients, although the difference between groups was not statistically significant. In general, the overall social support of these patients (roughly 74, ranging from 72 to 78 points) was comparable to a prospective cohort that followed cancer patients since their diagnosis, with an overall social support score at baseline of 80.9 and decreased to 72 after 2 years.^63^

Although differences between PA groups for cardiovascular and immune-related biomarkers were not statistically significant, patients who were more physically active generally had lower levels of inflammatory biomarkers. With regard to cardiac measures, heart rate was significantly lower in more active individuals, which is expected, considering that higher resting heart rate has been inversely associated with physical fitness.^64^ Interestingly, elevated resting heart rate has been associated with increased prospective risk of mortality and disease recurrence in cancer survivors,^65,66^ supporting the hypothesis that poor cardiovascular fitness, and related autonomic sequelae, may be important therapeutic targets in improving cancer-related outcomes. Our results also indicated that higher intensity of PA was associated with lower levels of inflammation, which corroborates recent evidence in cancer patients,^44-46^ and suggests that higher intensities of PA may be uniquely desirable to mitigate cancer- and treatment-related chronic inflammation in patient populations.

In a multivariable analysis, we also examined correlates of QoL, with a regression model indicating that age, fatigue, anxiety, and social support were the strongest independent correlates, explaining 71% of the variance in QoL. However, when added to the model with the other correlates, PA was no longer significantly associated with QoL, though this may have been related to the reduction in total sample size given missing data of other correlates entered in our model. Our findings are supported by a matched cohort study with breast cancer survivors, where the authors identified age as a significant predictor for general QoL assessed by the SF-36 questionnaire.^67^

This study has strengths and limitations. One strength is that we were able to assess the individuals enrolling in the InspireHealth clinic as a whole, including sociodemographic, health history, physiological and psychological characteristics, and health-related behaviors. Our main objective was to describe patients who seek integrative care, and despite issues related to selection bias, we were able to do so. Nonetheless, our study had limitations, as well. The study design was cross-sectional, thus limiting causal analysis or interpretation. The sample was drawn by convenience; and therefore, it did not include cancer patients not seeking integrative care. Because all patients shared a similar interest in CIM, our results should be interpreted with caution and not necessarily extrapolated to the broader population of cancer patients. There is a possibility that patients with better QoL make time to exercise more often than those with poorer QoL, which may be a prediagnosis difference that persisted throughout their illness. Moreover, the InspireHealth clinic is located in Canada, and this is also a limiting factor in generalizing our findings to populations of other countries.

Regarding our analyses, there were some missing data, and such a limitation might have negatively affected power in the analyses, including the regression model. The instrument used to capture PA was a self-reported questionnaire. Although commonly used to capture leisure-time PA, we are aware of existing discrepancies between self-reported and objectively measures of PA.^68,69^ Other variables were also self-reported; for example, the use of medications and supplements, and its open-ended format allowed patients to omit identifying prescribers. The missing information accounted for 41.9% of all pharmacologics and 69.7% of all supplements. Furthermore, errors in memory recall could have biased these and other self-reported variables. Additionally, our physiological biomarker analyses were unadjusted for additional confounders, such as type of drug, stage of the disease, current treatment, depression, and anxiety. Finally, we did not analyze eating patterns or diet in our study, which is also an important health behavior.

## Conclusion

We observed significant differences in self-reported physical and functional well-being, level of fatigue, physical symptoms associated with cancer, the prevalence of depression, and the use of supplements among patients who engaged in more versus less PA postdiagnosis. We observed positive associations between total and moderate PA and QoL, as well as negative associations between vigorous PA and inflammation. Gender, fatigue, anxiety, and social support were strong correlates of QoL. Considering the continuous growth of integrative care use globally,^70-73^ and especially in Canada,^74^ our findings can inform health services and decision-makers how to optimize physical and functional well-being in cancer patients undergoing active treatment.
